# The Innovative Biomaterials and Technologies for Developing Corneal Endothelium Tissue Engineering Scaffolds: A Review and Prospect

**DOI:** 10.3390/bioengineering10111284

**Published:** 2023-11-03

**Authors:** Miaomiao Chi, Bowei Yuan, Zijun Xie, Jing Hong

**Affiliations:** 1 Department of Ophthalmology, Peking University Third Hospital, Beijing 100191, China; chimiaomiao01@stu.pku.edu.cn (M.C.); 2116391081@bjmu.edu.cn (B.Y.); xzjldbld@pku.edu.cn (Z.X.); 2 Key Laboratory of Vision Loss and Restoration, Ministry of Education, Beijing 100191, China

**Keywords:** corneal endothelium tissue engineering, biomaterials, drug delivery, surface topography, innovative technologies

## Abstract

Corneal transplantation is the only treatment for corneal endothelial blindness. However, there is an urgent need to find substitutes for corneal endothelium grafts due to the global shortage of donor corneas. An emerging research field focuses on the construction of scaffold-based corneal endothelium tissue engineering (CETE). Long-term success in CETE transplantation may be achieved by selecting the appropriate biomaterials as scaffolds of corneal endothelial cells and adding bioactive materials to promote cell activity. This article reviews the research progress of CETE biomaterials in the past 20 years, describes the key characteristics required for corneal endothelial scaffolds, and summarizes the types of materials that have been reported. Based on these, we list feasible improvement strategies for biomaterials innovation. In addition, we describe the improved techniques for the scaffolds’ surface topography and drug delivery system. Some promising technologies for constructing CETE are proposed. However, some questions have not been answered yet, and clinical trials and industrialization should be carried out with caution.

## 1. Introduction

The cornea is a transparent and avascular tissue located in the anterior part of the eye, accounting for more than two thirds of the total refractive power. The cornea has five layers, the corneal endothelium being the single layer of cells that is positioned on the back of the cornea. With a leakage barrier and ionic pumps, this cell layer controls the cornea’s ionic concentration and water content, maintaining the cornea’s semi-dehydrated condition and preserving its normal thickness and transparency. There are many causes for the loss, disorganization, and destruction of the corneal endothelium. Since human corneal endothelial cells (hCECs) do not have a regenerative ability in vivo, the natural spread and migration of the remaining hCECs serve to compensate for the loss of the corneal endothelium [1]. However, the corneal endothelium becomes dysfunctional when the corneal endothelial cell density (CED) decreases to less than 400 CECs/mm^2^, which leads to bullous keratopathy, a kind of corneal edema. This condition eventually results in vision loss and is called corneal endothelial blindness [2].

The only available treatment for corneal endothelial blindness is corneal transplantation. Corneal endothelial dysfunction is the primary cause of corneal transplantation [3]. Current standard surgical procedures include penetrating keratoplasty (PK) and endothelial keratoplasty (EK). The latter includes Descemet’s stripping automated endothelial keratoplasty (DSAEK) and Descemet’s membrane endothelial keratoplasty (DMEK), both of which involve transplanting healthy monolayer hCECs from a cadaveric donor into the patient’s anterior chamber. DMEK transplants only the Descemet’s membrane (DM), while DSAEK transplants the DM and a part of the stroma. The layer is then attached to the posterior surface of the cornea with air-filled material. Nowadays, EK has become the preferred surgical method to replace PK due to its smaller invasiveness, better postoperative vision, and lower risk of immune rejection [2].

**Figure 1:**
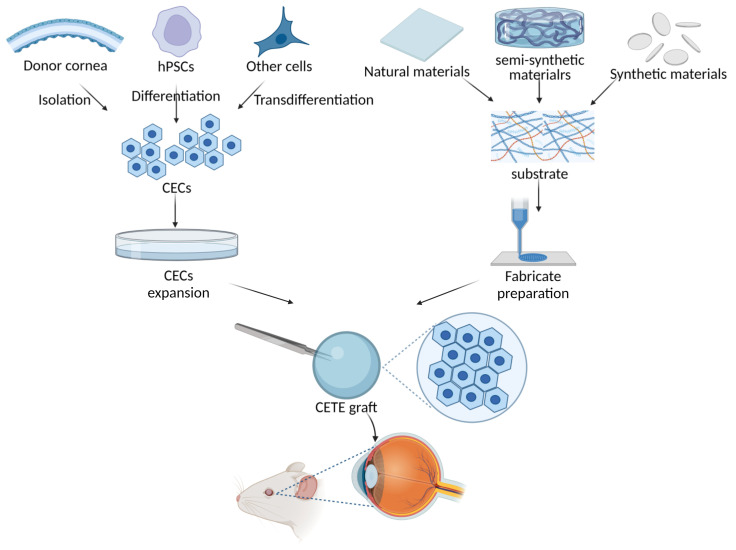
Scheme of corneal endothelial tissue engineering (CETE) constructed based on scaffolds and seed cells. Human corneal endothelial cells (hCECs), as the seed cells, can be derived from donor corneas isolation, human pluripotent stem cells (hPSCs) differentiation, or other types of cells transdifferentiation. Increased hCECs can be obtained by expansion in vitro. The source of scaffolds includes natural, synthetic, or semi-synthetic materials. The scaffold is fabricated through techniques including 3D bioprinting and electrospinning. Finally, hCECs are loaded onto the scaffold to create a CETE graft. These grafts can then be inserted into the cornea’s posterior surface of an animal for research purposes with the eventual goal of transplanting into humans.

Unfortunately, due to the global shortage of donor corneas, less than 1.5% of patients in need of corneal transplants are able to undergo allogeneic transplants. The number of patients in need of treatment far outweighs the number of available donors by a ratio of 70:1 [3]. Therefore, there is an urgent need to discover a corneal endothelium substitute to resolve this problem. Regenerative medicine based on CECs cultivation and expansion in vitro has become an emerging research field. Cell injection and scaffold-based corneal endothelium tissue engineering (CETE) are the two main research avenues. In addition to addressing the worldwide shortage of donor corneas, cell injection therapy may prove to be less invasive and more tolerable than endothelial keratoplasties or penetrating keratoplasty due to a lower risk of rejection. Controlling the quantity of CECs injected and the destiny regression are challenging aspects of cell injection. Too few cells will produce a final monolayer with a low cell density that is ineffective at relieving corneal edema, whereas too many cells or unadhered cells may block the trabecular meshwork, preventing the outflow of aqueous humor and elevating intraocular pressure [4]. Furthermore, the therapeutic application of cell injection therapy is significantly constrained by the need for a fully complete DM. Jodhbir S. Mehta’s team have demonstrated that the injected CECs do not form a functional monolayer and are unable to improve corneal transparency in a rabbit model of corneal damage when directly injected onto the bare stroma after DM removal [5,6]. As shown in Figure 1, CETE is constructed based on scaffolds and seed cells. Human corneal endothelial cells (hCECs), as the seed cells, can be derived from donor corneas isolation, human pluripotent stem cells (hPSCs) differentiation, or other types of cells transdifferentiation. Increased hCECs can be obtained by expansion in vitro. The source of scaffolds includes natural, synthetic, or semi-synthetic materials. The scaffold is fabricated through techniques including 3D bioprinting and electrospinning. Finally, hCECs are loaded onto the scaffold to create a CETE graft. These grafts can then be inserted into the cornea’s posterior surface of an animal for research purposes with the eventual goal of transplanting into humans. Long-term success in CETE transplantation may be achieved by selecting the appropriate biomaterials as scaffolds of corneal endothelial cells and adding bioactive materials to promote cell activity.

This article reviews the progress in bioactive materials for CETE in the past 20 years. The key properties required for corneal endothelial scaffolds are outlined. On the basis of a summary of the substrate materials that have been reported in recent investigations, feasible biomaterial advances and improvement strategies are listed here. We summarize improved techniques for the scaffold’s surface morphology and drug delivery system. Finally, some promising technologies for constructing CETE are proposed.

## 2. Key Properties Required for Corneal Endothelial Implantation Substrate

**Figure 2:**
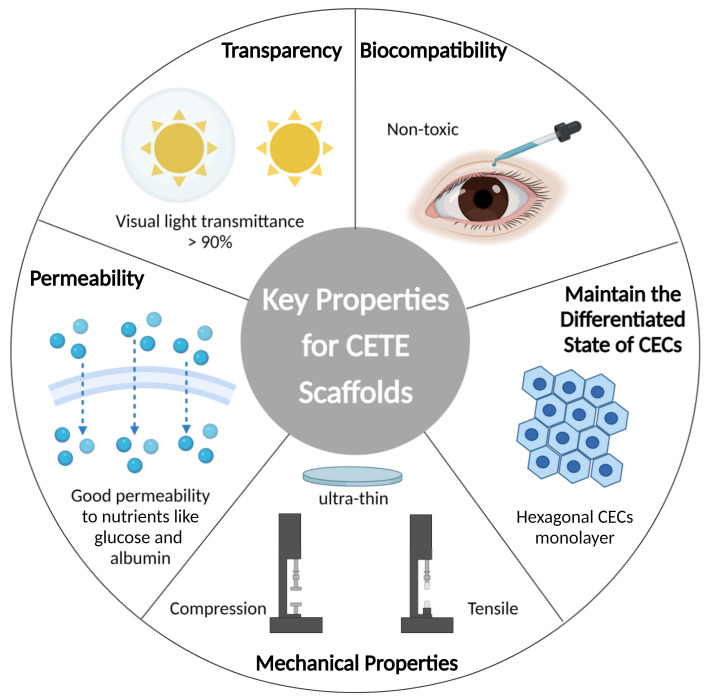
Schematics of key properties for CETE scaffold. Imitating the native Descemet’s membrane, the scaffold acts as an extracellular matrix (ECM), providing suitable microenvironment and signal support for the behavior of CECs. Transparency, nutrient permeability, proper mechanical strength, biocompatibility, and ability to maintain the differentiated state of CECs are some of the specific characteristics.

The implantation scaffold serves as the extracellular matrix (ECM), providing the appropriate microenvironment and signaling support for CECs. DM is secreted and formed by CECs. Collagen IV is the basis of DM, which also includes laminin, fibronectin, collagen VIII, and perlecan, and plays an important role in the development of corneal endothelium. As shown in Figure 2, it is crucial to keep the performance characteristics of the scaffold as close to native DM as possible. A few parameters can be used to assess the implant’s qualities, ensure the successful replacement’s implantation, and optimize the morphology and functionality of endothelium.

### 2.1. Transparency

Transparency is connected to the corneal endothelium’s ability to transmit light, which enables vision of the endothelium. DM is highly transparent (90%) in the visual range [7]. Ultraviolet–visible (UV-VIS) spectroscopy is the preferred technique for determining transparency, measuring continuous spectra in the visual area (380–750 nm). Compared with other materials, the amniotic membrane and silk fibroin perform poorly. It is recommended to avoid using electrospinning as a processing technique as its transparency is poor and scattering occurs due to the presence of a large number of randomly oriented polymer fibers in the scaffold [8]. The scaffold must have a refractive index as similar as possible to that of the natural cornea, and a sufficient CED to maintain normal corneal transmittance. The natural cornea contributes significantly to the eye’s refractive power and has a refractive index of 1.367. Refractive errors may result from the implant’s uneven attachment to the cornea’s posterior surface.

In addition, a sufficient CED on the scaffold creates tight junctions (normal CED is 2500–3000 cells/mm^2^), which maintains corneal transparency through barrier and Na+/K+-ATPase pump functions.

### 2.2. Biocompatibility

The ability of living organisms (hosts) to react to inert materials is known as biocompatibility. First and foremost, the substrate and its degradation products must be non-toxic. In vitro cytotoxicity tests and further in vivo tests should be conducted on the materials. The U.S. Food and Drug Administration (FDA) has authorized numerous synthetic materials, including PEG, poly (lactic acid) (PLA), and poly (lactic-co-glycolic acid) (PLGA), for use in biomedical applications.

The CETE scaffold should adhere constantly to the posterior surface of the cornea and sustain CECs adhesion. Hydrophilic materials (e.g., gelatin, chitosan, and collagen) can support adhesion to the cornea while promoting cell adhesion without the use of additional adhesives [9,10]. Long-term adhesion between the scaffold and the cornea during the healing process is achieved through bioactive protein adsorption, mainly fibronectin, laminin, and collagen [11,12]. The shape of the scaffold has a similar curvature to the corneal stroma, which helps to adhere perfectly to the posterior surface of the cornea. However, poly(ε-caprolactone) (PCL), poly (D, L-lactic acid) (PDLLA), and other hydrophobic materials have low protein fouling [13]. Some surface modification or ligand conjugation methods are generally adopted to promote cell adhesion, such as adding carboxylic acid and amine groups and binding collagen or other adhesion ligands.

### 2.3. Mechanical Properties

Mechanical property is a scaffold’s capacity to hold its shape and resist deformation. The scaffold is inserted into the anterior chamber via a tiny intraocular incision after being physically stretched and folded by tools. During this process, the scaffold should maintain its physical and optical properties. The scaffold must support intraocular pressure and serve as a barrier at the same time since it has adequate mechanical strength and elasticity. The stiffness of the scaffold can significantly affect the behavior of the attached cells. Research has reported that the tensile modulus of DM is about 2.6 MPa [14]. Therefore, the scaffold’s stiffness should be close to that of DM to provide a microenvironment favorable to the extension of natural CECs. The most reported parameters for the quantitative measurement of mechanical property include the Young’s modulus and the stress and strain at break, using a tensile testing apparatus [8]. The Young’s modulus is expressed as a change in force per area divided by the change in strain. The stress and strain at break accurately represent the stresses that a scaffold can withstand and the probability of tearing. Nevertheless, there is currently no unified standard for the detection methods of the mechanical properties of the scaffold. It should be noted that there are varying values due to no unified standard for the methodology employed. Both different techniques and the hydration state affect the final detection result [15].

The thickness of natural DM ranges from 2 to 10 μm. The implant thickness for DSEK, the most popular kind of corneal endothelial transplantation, is between 100 and 150 μm [16]. The proper thickness of the implant allows for easy insertion into the anterior chamber through a small incision. Clinically, ultrathin grafts (between 10 and 20 μm) for DMEK are strongly supported because they reduce the likelihood of refractive aberration [17]. The thickness of the scaffold should be measured to ensure the refractive power and transmittance abilities of DM. However, due to differences in detection methods and substrate hydration status, the results of substrate thickness from various sources are not accurate. The detection methods include scanning electron microscopy (SEM), optical coherence tomography (OCT), interferometry, etc. It is recommended to determine the thickness of the implant in a hydrated state to better reflect the conditions in the eye. Additionally, the water-hydrated state of the tested scaffold is not specified.

### 2.4. Permeability

Permeability refers to the ability to permit the passage of nutritional elements through the CECs. Since the cornea has no blood vessels, nutrients (mainly glucose) are mostly obtained from the aqueous humor through the CECs. Therefore, the scaffold must have good permeability to nutrients and biomolecules like glucose and albumin. The diffusion coefficient of glucose through the scaffold can be used to evaluate the permeability of the scaffold. The human corneal diffusion coefficients of glucose and albumin are (2.6 ± 0.3) ×10^−6^ cm^2^ s^−1^ and (1.0 ± 0.2) ×10^−7^ cm^2^ s^−1^, respectively [18]. It is important to note that the diffusion of nutrients by the scaffold may be affected by the CECs barrier.

### 2.5. Ability to Maintain the Differentiated State of CECs

In addition to physical and biochemical properties, cell response reflects the possibility of the material to act as a decent endothelial cell scaffold. Cell morphology is the simplest and most accessible evaluation criterion. CECs achieve monolayer fusion and show a hexagonal morphology when cultured on a scaffold [19].

Due to the lack of specific surface markers, previous research mostly used Na+/K+-ATPase, ZO-1, and N-cadherin to verify CECs. The expression of these proteins decreases when CECs age or undergo endothelial-to-mesenchymal transition (EndMT) [20]. The functions of CECs can be further demonstrated through testing in vitro. Trans-endothelial electrical resistance (TEER) and an Ussing chamber are used to detect electrical potential over the endothelium, which can reflect the ionic permeability of CECs [21,22].

## 3. Substrate Materials

The CETE scaffold can be derived from membranes or films which are isolated from the ECM of natural sources (e.g., decellularized corneal stroma, amniotic membrane, lens capsule, etc.). On the other hand, using polymers to make a completely new scaffold is another choice, and consists of natural (e.g., collagen, gelatin, etc.), synthetic (e.g., PCL, PDLLA, etc.), and semi-synthetic (e.g., blend of chitosan with PEG, etc.) polymers. The advantages and limitations of each material for a CETE scaffold are summarized in Table 1.

### 3.1. Natural Tissue

#### 3.1.1. Decellularized Corneal Stroma

After the decellularization process, human- or other animals-derived corneas are removed from their original cells, immunogenic compounds, and infection factors, which can be used as CETE scaffolds. Human-derived decellularized corneal stroma retains appropriate mechanical properties as well as natural recognition signals that promote cell adhesion, migration, and proliferation. This is an ideal source of substrates, but its clinical application is limited by the shortage of donors. The use of femtosecond laser cutting has been reported to obtain six ultrathin implants from a single corneal donor [42].

Decellularized porcine corneal stroma is similar to that of human origin, with better dimensional and optical properties, and is less restricted by donor sources. However, existing decellularization methods cannot completely remove all traces of cells. As the most important xenoantigen in pigs, a-gel can trigger a strong immune response in humans resulting in rejection. To address this problem, some scholars have attempted to improve the decellularization procedure by introducing CRISPR-CAS9 technology [43].

#### 3.1.2. Descemet’s Membrane

Descemet’s membrane is composed of collagen and other substances secreted by CECs, and increases in thickness over time, from 3 μm at birth to 13 μm in old age. DM is the most ideal choice for constructing CETE since it is the natural substrate for CECs. However, this choice has drawbacks, such as insufficient donor sources, being too thin to operate, and the danger of infectious diseases transmission. Despite being successful in the cat model [24], porcine-derived DM also has a risk of xenograft rejection.

#### 3.1.3. Amniotic Membrane

Human amniotic membrane (AM) is a natural and inert substance with high biocompatibility and low immunogenicity. It possesses excellent antibacterial, anti-inflammatory, anti-fibrosis, and anti-angiogenesis characteristics, and is widely used in ophthalmology. The use of AM as a scaffold, fabricated by decellularization and lyophilization techniques, has shown some promise in primate models [26]. However, the semi-transparency of AM limits its application. In addition, studies have found its low mechanical properties, unpredictable biodegradation rates, donor heterogeneity, potential for granulomatous reactions, and risk of contamination and infection transmission [27]. Research has been conducted to improve the properties of AM through surface coating and chemical cross-linking. For instance, one study reported using cross-linked AM scaffolds to improve the mechanical properties of corneal endothelial regeneration [25]. However, there is no solution to the unpredictable biodegradation rates.

#### 3.1.4. Decellularized Human Lens Capsule Membrane

The collagen IV and laminin-based human lens capsule membrane shares many characteristics with the DM, including transparency and biocompatibility. It makes sense to use this wasted material in cataract surgery because the anterior lens capsular membrane is striped during the procedure. Using this material as a substrate, studies have discovered endothelial cell adhesion and the formation of multiple cell interconnections between growing CECs [28]. However, there are some disadvantages: dependence on the donor and the small diameter of the available anterior capsular membrane.

#### 3.1.5. Decellularized Fish Scales

The structure of fish scale is composed of multiple layers of collagen I, and good optical, mechanical, and biological properties have been demonstrated in prior studies that were cultured with corneal epithelial and stromal cells in vitro. However, decellularized fish scale scaffolds do not work well for CETE, with CECs exhibiting high polymorphism and poor adhesion [30]; this result was improved following collagen IV, fibronectin, and laminin encapsulation, though [29].

### 3.2. Natural Polymers

Natural polymers exist in the cell membranes and ECM of different biological origins and are large molecules composed of proteins or polysaccharides. Common natural polymers include collagen, alginate, starch, chitosan, etc. Due to their natural sources, they offer great advantages, such as biocompatibility and binding domains. These structural domains help in cell adhesion and differentiation when recognizing a natural matrix [44].

Nevertheless, natural variability causes batch-to-batch variance in natural polymers, which causes variable properties among polymers even when they come from the same source. Natural materials have the possibility to transfer infections or elicit immunological responses. Their weak thermal and mechanical qualities and quick degradation rates limit their utility.

#### 3.2.1. Collagen

Collagen is the most important protein for the corneal, since collagen types I and IV are the main protein components in the cornea. Collagen has necessary properties for scaffolds, such as desirable biodegradability and biocompatibility. Vázquez et al. developed human purified type I collagen membrane cultured hCECs, which expressed characteristic markers of the corneal endothelium. Furthermore, the scaffolds-cultured rabbit CECs were transplanted into a rabbit, which helps corneas to maintain transparency for as long as 6 weeks without obvious edema or immune rejection [31]. The mechanical properties, however, are insufficient to withstand surgical operation. According to the published literature, mixing natural polymers (e.g., collagen) with a cross-linker (e.g., EDC/NHS) [45] or synthetic polymers helps to achieve a better mechanical property. In addition, removing excessive water through mechanical pressure can also overcome this drawback. Levis et al. described the first use of plastic compressed collagen as a highly effective, novel carrier for hCECs. The scaffold remains fully intact after surgical procedure, but the researchers did not provide a specific measurement date for the mechanical property [45].

#### 3.2.2. Gelatin

Collagen I is hydrolyzed to produce gelatin, which is more flexible and cost-effective than the former. Gelatin contains numerous functional amino acid sequences, including arginine–glycine–aspartic acid (RGD), which encourages cell adhesion and growth, and matrix metalloproteinase (MMP) used for cell remodeling. However, the disadvantage is that the mechanical properties of gelatin are too poor, so it must be cross-linked with cross-linkers (e.g., EDC/NHS) [10] or blended with synthetic polymers (e.g., poly(N-isopropylacrylamide) [46]. Niu et al. developed a heparin-modified gelatin cross-linked with EDC/NHS that improves the mechanical properties of gelatin, with a Young’s modulus of 3.5 ± 0.3 MPa, tensile strain at break of 57.7 ± 13.8%, and tensile stress at break of 1.4 ± 0.4 MPa. Additionally, the heparin-modified scaffolds had a greater capacity to absorb basic fibroblast growth factor (bFGF) and showed better release kinetics for up to 20 days, which improved hCECs survival and reduced cellular loss [10]. Although rabbits are often used as animal models for ophthalmic experiments, the rabbit corneal endothelial injury model is not convincing. Rabbit CECs have the ability to regenerate in vivo, and the postoperative effect may be affected by their own cells. The corneal endothelium of cats is similar to that of humans, which is not renewable in the body. With a deep anterior chamber, cats are more suitable for surgery [47]. Cats are economical and easier to obtain when compared to other animals (such as pigs and sheep).

#### 3.2.3. Hyaluronic Acid

Hyaluronic acid is a natural mucopolysaccharide present in the aqueous humor and vitreous body. It is one of the most often employed ocular biomaterials in clinical practice because of its intrinsic biocompatibility. For example, it is used as a viscoelastic agent in cataract surgery and deep lamellar corneal transplantation. However, hyaluronic acid must be cross-linked to overcome the problem of rapid dissolution in a liquid environment. Porous collagen and hyaluronic acid scaffolds modified with 1-ethyl-3-(3-dimethyl aminopropyl) carbodiimide (EDC) showed promising results [32].

#### 3.2.4. Silk Proteins

Silk and its derived silk proteins have superior mechanical strength compared to the aforementioned natural polymers. Cell adhesion is enhanced by coating silk fibroin (SF) with collagen IV. As Madden et al. reported, the cell attachment ratio and counts significantly improved compare to uncoated SF and FNC/laminin/chondroitin-coated SF [34]. The use of aloe vera gel to cross-link SF improves the scaffold’s flexibility and hydrophilicity, which facilitates the adhesion and expansion of CECs [35]. Kim et al. revealed that the critical morphology of CECs was formed on the AV/SF scaffold rather than SF alone through field emission scanning electron microscopy. They indicated that 3 wt % AV/SF increased the cell viability and maintained its functions well [35]. As a plasticizer, glycerin allows us to reduce the brittleness of a material by reducing the specific interactions between the protein subunits, resulting in an increase in chains mobility and film flexibility. According to Song et al., glycerin/SF films show a rougher surface with respect to the SF film. Furthermore, glycerin showed a decrease in material thickness from 10.39 μm (SF) to 7.25 and 6.37 μm (1, 3% glycerin/SF) [33].

#### 3.2.5. Chitosan

Chitosan, a naturally abundant cationic polymer, is chemically made of cellulose-based biopolymers derived by deacetylating chitin. It has good biodegradability, biocompatibility, and anti-microbial qualities and is therefore increasingly used in pharmaceutical and biomedical applications [48]. This substrate is prone to breakage after surgical operation, so it has to be combined with other materials for use. The composite membrane created by mixing chitosan and collagen and cross-linking with EDC has significantly improved optical transparency and mechanical strength. The tensile strength of the chitosan/collagen-blended scaffold was close to that of the human cornea (approximately 4 Mpa), showing no significant difference. Furthermore, both in vitro and in vivo tests have demonstrated that it has good permeability to both glucose and albumins [36].

### 3.3. Synthetic Polymers

When compared to natural materials, scaffolds made of synthetic polymers have certain unique advantages. Synthetic polymers are made up of monomers with varying lengths. By appropriately selecting the content of monomers and initiators and optimizing reaction conditions, it is possible to produce scaffolds with predictable structures and physicochemical properties. They can be an alternative to natural scaffolds due to their advantages of low cost, reproducibility, mass production, and customizability. However, the main shortcoming of synthetic materials is the absence of cellular recognition signals, which prevent cells from attaching and completely integrating with human tissues.

Poly (lactic-co-glutamic acid) (PLGA) is a hydrophobic polymer synthesized from poly (lactic acid) (PLA) and poly (glutamic acid) (PGA) that has been approved by the FDA for biomedical applications. While PLA is hydrophobic, PGA is hydrophilic. By changing the ratio of the two materials, the mechanical strength, swelling properties, and degradation rate of the blended membrane can all be modulated. Huhtala et al. tested different proportions of PLGA (50:50 PDLGA, 85:15 PDLGA) to culture bovine CECs. As the PLA concentration rises, the biocompatibility of the scaffold declines. This result may be due to the faster degradation rate causing the pH of the medium to become more acidic, ultimately leading to decreased cell survival [37]. Kim et al. used collagen-type-I-coated PLGA films (Col I-PLGAs) as a scaffold for rabbit CECs, adequately taking both mechanical properties and biocompatibility into account. Compared with bare PLGA films, modified Col I-PLGA film displayed good transparency and stability [49].

PCL is a semi-crystalline polyester with good biocompatibility, low toxicity, and biodegradability. PCL may be molded into many shapes because of its excellent thermal stability and flexible elasticity at room temperature. Additionally, its surface is easily modifiable. Despite its somewhat low mechanical qualities, PCL is frequently mixed with other polymers such as chitosan, PEG, etc. [9,44]. Kruse et al. fabricated PLGA and PCL electro-spun membranes to culture hCECs. Cells maintain the hexagonal morphology but do not form a uniform monolayer [50].

Polyethylene glycol (PEG) is a hydrophilic, neutral polymer of ethylene oxide. PEG is widely employed in tissue engineering because of its adaptability, amphiphilicity, capacity for hydration, and biocompatibility. Additionally, it has a very high degree of transparency [9], which makes it an excellent choice as a corneal endothelial scaffold. Ozcelik et al. fabricated PEG-based hydrogel films, on which sheep CECs proliferate with natural morphology and become 100% confluent within 7 days. After 28 days following surgery, the corneas reveal minimal inflammatory responses and no toxicity [9].

According to reports, other synthetic polymers have also been utilized. Bovine CECs were used to test polyvinyl alcohol (PVA), poly (ethylene-co-vinyl alcohol) (EVAL), tissue culture polystyrene (TCPS), and polyvinylidene fluoride (PVDF), with PVDF showing the greatest results. As a bioactive substrate, PVDF enabled bovine CECs to synthesize and stock more collagen IV of ECM, providing a better environment for bovine CEC culture [38].

### 3.4. Semi-Synthetic Polymers

Both natural and synthetic materials have their own advantages and disadvantages. The composition and properties of synthetic polymers can be artificially altered to a greater extent. Natural polymers may vary from batch to batch, which may change the final properties of the product. In terms of biological response, synthetic polymers are generally more inert, although the surface can be modified or functionalized through strategies. Natural polymers, however, have the appropriate amino acids and proteins required for cell adhesion and guidance, which may adversely affect the immune response.

The natural and synthetic materials can be combined to form a blend or chemically cross-linked with each other to create, e.g., a copolymer. Multiple material requirements must be met for CETE, including cytocompatibility, reproducibility, supply-chain simplicity, transparency, and ease of handling by the surgeon (desirable flexibility and adjustable characteristics). Therefore, semi-synthetic polymers could be able to satisfy all the requirements at once, improving the scaffold’s physical and biological qualities.

#### 3.4.1. Methacryloyl Gelatin

Methacryloyl gelatin (GelMA), which has strong biocompatibility and most of the functional amino acid motifs of gelatin (including RGD and MMP), is produced by the reaction of gelatin with methacrylic anhydride (MA). In addition, GelMA can undergo photo-induced chemical cross-linking reactions to increase mechanical strength and rigidity. Because of good temperature-sensitive properties, biocompatibility, and printability, GelMA has been widely used as a substrate component for hydrogels and bioinks co-cultured with cells [51,52].

In the field of cornea, a study has modified GelMA by developing a sequential hybrid cross-linking process (physical cross-linking followed by UV cross-linking) to create an improved material named GelMA+ [39]. In vitro and in vivo tests revealed that GelMA+ had an eight-fold higher mechanical strength and slower kinetics of breakdown than conventional GelMA. The hCECs grown on this scaffold expressed markers such as ZO-1 and maintained a good morphology and function. Recent studies have shown that CECs adhere firmly to the surface of poly-NAGA-GelMA (PNG) bioink with more than 90% viability and a well-maintained phenotype [53]. However, there are no studies to support its viability in vivo.

#### 3.4.2. Chitosan-Based Bioactive Materials

Many researchers have combined different types of chitosan-based membranes with other polymers to examine CETE. Studies that tested the functionality of ultrathin hydrogel membranes prepared from a hybrid of chitosan and PEG showed that the scaffold supported the attachment and growth of sheep CECs and worked effectively during in vitro surgery on sheep eyes [40]. Another study reported after co-culturing 25% PCL and 75% chitosan for 14 days that the CECs expressed the tight junction protein ZO-1, Na+/K+ ATPase, and connexin-43, confirming the functional features of the cells [54]. The team’s subsequent work found that collagen-IV-rich ECM could be secreted by hCECs cultured on blended membranes [41].

Although the majority of the previous studies have focused on the utilization of chitosan for the preparation of bioactive materials, some studies have explored the possibilities of chitosan derivatives. Hydroxyethyl chitosan is one of the commonly used derivatives of chitosan. A combination of gelatin, hydroxyethyl chitosan, and chondroitin sulfate was used to create a membrane, and its water content, ionic permeability, and glucose permeability were all extremely similar to those of a natural cornea [55].

The utilization of bioactive components to tune and functionalize the material surface was another area of study emphasis. Chitosan can be treated with a range of anionic polymers because the main amines on the skeleton chain of the material help to create polycations (following protonation in acidic conditions) [56].

## 4. Materials Innovation

### 4.1. Peptide Hydrogels

Hydrogel materials have a distinct edge over other materials, with high 3D cross-linking and a resemblance to natural ECM. Polypeptide hydrogel is one of the hydrogels with unique advantages. In addition to having the biocompatibility, bioactivity, and degradability of natural macromolecular hydrogels, polypeptide hydrogels also have the adjustable, customizable, and repeatable mechanical properties of synthetic polymer hydrogels.

Research has shown that a viable substrate for CECs amplification and transplantation is a peptide hydrogel made of poly-ε-lysine (pεK) that has been cross-linked with octanedioic acid [57]. The scaffold is thin, transparent, porous, and robust. Furthermore, the study experimented with CECs from human and porcine. Interestingly, it found that, unlike human CECs, porcine CECs only adhere to scaffolds with the cell-binding peptide, RGD. This result suggests that p ε K hydrogels can be tailored by covalent binding RGD to provide a surface for CEC attachment and growth.

At present, well-established peptide sequences for use in hydrogel scaffolds include EAK16 and RADA16, MAX1 and MAX8, elastin-like polypeptides (ELPs), etc. [58]. The properties of peptide hydrogels have been optimized by changing the composition and structure (e.g., amino acid type and number), adding functional sequence peptides (e.g., cell adhesion peptides RGD, IKVAV, and YIGSR), or incorporating other polymers (e.g., PEG) or biomolecules (e.g., proteins, enzymes) [58]. The advent of novel 3D bioprinting has realized the accurate control of the intricate spatial geometry of peptide hydrogel scaffolds, thus enabling them to resemble the native ECM environment. However, there is limited research on peptide hydrogels in the field of CETE or even cornea.

### 4.2. Injectable Hydrogels

The primary benefit of hydrogels is their injectability, minimal invasiveness, and availability for irregularly shaped sites [59]. A mixture of polymer/monomer solution (precursor) and therapeutic agent is placed into the injection. Due to the low viscosity, it can be injected into the targeted site of the body through a syringe needle. After that, the therapeutic agent-carrying hydrogel is then formed by a physical or chemical cross-linking reaction, the viscosity of which increases dramatically during the phase change from a sol to a gel.

Injectable hydrogel systems include natural, synthetic, and hybrid polymers and their cross-linking reactions and conditions that induce gel formation. They exhibit special benefits in the area of tissue-engineered corneas since they serve as scaffolds to provide space for cell survival and as carriers that enable the encapsulation and delivery of bioactive molecules like drugs and proteins. Studies have reported that CECs were successfully encapsulated in a composite hydrogel composed of chitosan, hydroxypropyl chitosan (HPCTS), and sodium alginate dialdehyde (SAD) and injected into the anterior chamber to repair the endothelium in situ. The gel-encapsulated CECs could survive and maintain normal morphology on native DM [60].

However, the mechanical properties of injectable hydrogels are still poor. Controlling the rheology is challenging, and the injection process might be troublesome due to rapid sol–gel transitions [61].

### 4.3. Functional Nanomaterials

Functional nanomaterials refer to nanomaterials with various biological and chemical activities, such as gold nanoparticles, iron oxide nanoparticles, carbon nanomaterials, etc., which have different structures and sizes and have certain applications. Nanomaterials are widely used in the treatment of ophthalmic diseases, and previous related studies have focused on the nanomaterial delivery of drugs, such as gold nanoparticles (GNPs) and superparamagnetic iron oxide nanoparticles (SPIONs).

With the rapid development of regenerative medicine in recent years, nanomaterials applied to tissue-engineered corneas have been proven to be promising. Functional nanomaterials can be used as reinforcement to improve the performance of corneal endothelial scaffolds. Carbon quantum dots (CQDs) have been reported to have applications in ocular nanomedicine [62]. According to the findings, positively charged CQDs derived from glucosamine hydrochloride and spermidine could effectively enhance the permeability of glucose and act as permeation enhancers for corneal endothelial therapy.

### 4.4. Exploration and Innovation in Other Materials

One class of intelligent materials that can adapt to changes in the environment are shape memory polymer (SMP) materials. They have the ability to "memorize" one or more preset shapes and can then be shaped into different temporary shapes as needed. They can instantly revert to their previous shape when specific conditions, such as temperature, an electric or magnetic field, light, humidity, or pH, are fulfilled. The SMP materials have much potential for application in CETE, as they can be modified to fit intricate corneal grafting techniques and then adhere nicely to the cornea’s posterior surface.

Conductive hydrogels are receiving more and more interest in the field of tissue engineering due to their exceptional electrical conductivity and biocompatibility. Conductive materials can regulate cell transmembrane activity and the transmembrane distribution of charged ions in electrically excited cells like neurons with the aid of non-invasive electrical stimulation technology, leading to the secure and effective regeneration of corneal nerves. In addition, conductive scaffolds can also regulate the release of some small-molecule drugs or factors, improving the therapeutic effect through synergistic effects. However, the mechanism by which conductive materials work in vivo is still unclear and needs further study.

## 5. Surface Topography

### 5.1. Surface Topography of the DM

**Figure 3:**
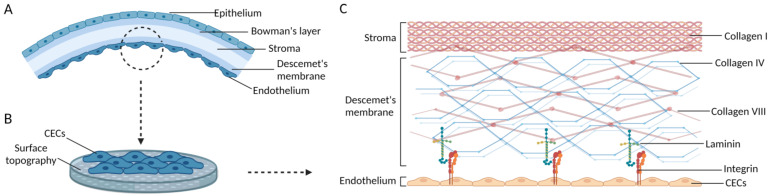
Schematics of the adhering hCECs and the ECM structure of Descemet’s membrane. (**A**) Five layers of the corneal tissue. (**B**) The ECM of Descemet’s membrane provides suitable surface topography for hCECs. (**C**) The ECM structure of Descemet’s membrane. Images are created by Biorender.com.

As shown in Figure 3, the main components of the ECM are collagen IV and collagen VIII, both of which are created by CECs. The main ECM skeleton in the DM is composed of a distinctive hexagonal arrangement of collagen VIII. Strong structural connections exist between CECs and their surrounding cells, and their basal side is adherent to the DM in a dendritic extension. Integrins’ extracellular domains bind to collagen, nestin, fibronectin, laminin, and other matrix components.

The topography of peeling DM has been observed by researchers employing 3D confocal microscopy [63]. Flat hexagonal pits with a maximum web height of 1 mm and a width ranging from 10 to 20 mm made up the microtopography. The unique hexagonal combs were irregularly shaped and exhibited a sinusoidal cross section.

In order to improve the behavior of adhering cells by providing a surface of specific roughness that controls the interaction between cells and matrices, scaffolds should closely mimic the surface topography of ECM [33,64]. Surface topography can be added to change interface properties, including hydrophilicity, surface energy, and cell interaction, without affecting the matrix material’s original performance characteristics [65].

### 5.2. Effect of Adding Surface Topography on CECs

Studies have demonstrated that CECs can perceive biomechanical variations in their surrounding environment and regulate ECM formation during physiological and pathological processes [66]. The dynamic, reciprocal interactions between CECs and their surroundings have been described using mechanotransduction signaling mechanisms. This procedure allows for the conversion of biophysical cues from the ECM into intracellular biochemical signals that cause cellular responses. Surface topographical cues can affect the structure of the cytoskeleton through the JNK-ERK1/2 and PI3K pathways [11]. A recent review also provided a more in-depth discussion on the impact of nano textured scaffolds on corneal endothelial cell culture [67].

Many studies have confirmed the beneficial effects of surface topography on CECs growth [68]. The scaffold, created with a micro- and nanoscale patterned structure using GelMA [28], demonstrated how the behavior of CECs is directly impacted by the grating’s height and width. Specifically, compared to unpatterned structures, 1 mm columnar gratings with square and hexagonal morphology displayed a higher expression of Na+/K+-ATPase, and ZO-1, and better CED, homogeneity, and hexagonal morphology. The nanofiber structure created by electrospinning poly (sebacate glyceride) (PGS)-PCL exhibits a hexagonal morphology on the surface, which is beneficial for CEC growth [69].

In addition to the effects on endothelial cells, studies have shown that the addition of surface topography can modulate protein adsorption [70]. Some research examined the adhesion of proteins to the surface of patterned hydrogels, including bovine serum albumin (BSA), bovine fibronectin (FN), and bovine vimentin (VN) [71]. Although the smooth surface of substrates is non-adhesive, as expected, imprinted topography promoted cell adhesion and spreading. Specifically, both bovine FN and bovine VN preferred to adhere to the groove walls on the surfaces with line patterns. The researchers attributed the peculiar cell behavior to protein adsorption and geometry-dependent cytoskeletal arrangements.

## 6. Drug Delivery Strategies

### 6.1. Drug-Eluting Biomaterials

Most biomaterials employed as scaffolds in CETE are hydrophilic and have porous structures. Some bioactive molecules can be trapped inside these scaffolds, consequently allowing for continuous release over time and restriction in specific spaces.

Gelatin has an excellent water absorption capacity and porous reticular structure. After being coupled with heparin, gelatin exhibits a greater adsorption of basic fibroblast growth factor (bFGF) and better release kinetics for up to 20 days, which supported hCECs survival and expansion [10]. Similarly, dorzolamide hydrochloride mixed with PCL significantly reduced intraocular pressure in patients with high IOP [72].

Drug-eluting biomaterials generally release their contents mostly through diffusion, although controlled drug release can be accomplished by adjusting the grid’s deterioration, swelling, and mechanical deformation. Furthermore, supramolecular materials, DNA nanomaterials, and self-assembling amphipathic peptides are examples of innovative biomaterials that have been used in the non-ocular field. These promising research studies will help to improve the functionality and responsiveness of biomaterials and optimize targeted drug delivery.

### 6.2. Surface Modification

After introducing free radicals or functional groups to the surface of scaffold materials, they can covalently bond with biological active macromolecules like heparin and albumin. Earlier studies have investigated the effectiveness of the surface modification of CETE scaffolds. The utilization of components like collagen IV, fibronectin, and chondroitin sulfate laminin to coat the silk fibroin substrate in order to improve CEC adhesion suggests that collagen has the best effect after processing [34]. Polylysine can also be modified with synthetic RGD and other peptides to promote CECs behavior, while their binding with natural proteins shows enhanced cell adhesion and functionality [59].

Most of the aforementioned experiments aimed at improving biocompatibility and cell adhesion. However, some studies have explored biological functional molecules. Lysophosphatidic acid (LPA), an endogenous glycerophospholipid signaling molecule, has been reported to stimulate the growth of fibroblasts, keratinocytes, and endothelial cells by affecting cell adhesion, proliferation, and migration. Modifying silk fibroin membranes with LAP achieved a higher cell activity and phenotype [73]. β-carotenoids were also added to the surface of silk fibroin to promote the proliferation of endothelial cells [74]. In the future, more attention should be paid to bioactive molecules that support CECs function and in vivo validation should be carried out.

### 6.3. Nanoparticle Delivery

In order to achieve near-zero-order kinetics rather than burst release, nanoparticle delivery carrier systems have been developed for a better sustained release effect. Commonly used drug delivery carrier structures include nano (particles, micelles, tubes, etc.), liposomes, hydrogels (microspheres, sponges, etc.), membrane-controlled (coatings, emulsions, osmotic pumps, etc.), skeletons, etc. The most suitable carrier structure can be selected according to the type of drug to be delivered.

Research reported that the biodegradable PLGA microspheres prolonged the time of sustained release to 7–10 days when encapsulating the Rho-kinase (ROCK) inhibitor Y–27632 [75]. Some advanced techniques, such as electrospinning, provide assistance in the preparation of nanoparticles. The researchers prepared a cytocompatibility nanofiber synthetic film by electrospinning poly (ethylene terephthalate) (PET), which was seeded with ARPE-19 cells on the surface and loaded with electrospray-modified PLGA or polyglycolic acid (PGA) nanoparticles. The results showed that PLGA nanoparticles were released for 2 weeks while PGA nanoparticles were released for 1 day, achieving a delayed release of bioactive molecules. The scaffold has a dual function of delivering cells to the desired area while delivering drugs/factors to the eye for a long-term period [76]. However, because electrospray involves the use of compounds dissolved in solvents, care must be taken when encapsulating the bioactive fraction to ensure that its activity is not compromised.

## 7. New Technologies for Scaffold Preparation

### 7.1. Electrospinning

Electrospinning, a technique for fabricating ultrathin three-dimensional (3D) scaffolds of nanofibers, mimics the ECM microenvironment used for tissue engineering [77]. Unlike conventional densely arranged two-dimensional (2D) nanofiber membranes, 3D electrospun nanofiber scaffolds strive for more precise spatial control, endowing the scaffolds with sufficient porosity and ECM environmental settings as well as optimized properties (e.g., injectability, compressibility, and bioactivity) [78]. Furthermore, the 3D morphology can modulate cellular interactions and mediate cell growth, migration, and differentiation.

The differences between the structure, function, and application of the scaffolds were determined by the electrospinning material and method used, as well as the post-processing technology of the electrospinning scaffolds. A wide range of natural, synthetic, or semi- synthetic polymers dissolved in appropriate solvents can be electrospun to create 3D porous scaffolds in tissue engineering [78]. The direct preparation methods for electrospinning are categorized as multilayering electrospinning, sacrificial agent electrospinning, wet electrospinning, and ultrasound-enhanced electrospinning. Post-processing techniques are used to fine-tune morphological and physicochemical properties, including gas foaming, ultrasound, short fiber assembly, combining bioprinting, electrospraying, etc.

Kruse et al. applied electrospinning technology to construct CETE for the first time in 2018 [50]. Electrospinning was carried out using PMMA, PLGA, and PCL with the same parameters. PMMA was cytotoxic, whereas the biodegradable PLGA and PCL electrospun scaffolds were not. Only the morphology of hCECs on PLGA scaffolds showed a hexagonal shape. Compared to conventional solvent casting, electrospun PLA and poly (vinyl alcohol) (PVA) displayed a good folding resistance, stable release rate, and unobserved cytotoxicity, making it a potential ocular drug delivery carrier [79] (shown in Table 2).

On the basis of this research, scholars have prepared scaffolds with a more optimized performance by mixing synthetic polymers with natural polymers. Electrospinning nanofibers of silk fibroin/poly (L-lactic acid)-ε-caprolactone (P (LLA-CL)) were tested. The SF: P (LLA-CL) scaffold with a 25:75 mixing ratio showed the best transparency and highest cell proliferation [84]. Compared to pure PCL, the performance of PCL/collagen, PCL/gelatin, and PCL/chitosan-blended nanofiber scaffolds has been enhanced, resulting in better endothelial cell growth [81]. In addition to controlling fiber morphology, electrospinning can set the fiber orientation of the scaffold to function effectively. The 3D nanofiber scaffold was prepared using a novel electrospinning method. Due to its hemispherical and radial arrangement characteristics, the scaffold can guide the direction of the main collagen and actin filaments in the extracellular matrix, providing a favorable environment for corneal cells.

However, electrospinning technology cannot allow for a precise control of the network structure. If electrospun fibers are excessively dense, they may reduce light transmission in the scaffold, where transparency is critical. Light transmission can be improved by shrinking the fiber diameter, making the scaffold thinner, and using polymers with refractive indices that match those of the human cornea to maximize scaffold transparency [82].

### 7.2. Bioprinting

Three-dimensional (3D) printing, also known as additive manufacturing, is driving major advancements in the field of medicine. A wide range of tissues, including multilayered skin, bone, vascular grafts, tracheal splints, heart tissue, and cartilaginous structures, have already been produced with this technology and transplanted into body. Up to date, research on corneal bioprinting has mainly focused on the corneal epithelium and stroma, whereas studies on the corneal endothelium are still scarce [85,86].

A study explored the use of 3D bioprinting to generate implants that were transplanted into a rabbit model of endothelial injury [87]. Primary human corneal endothelial cells transfected with RNase5 siRNA were added to gelatin-based bioink that contained 0.02% RGD. The bioink was deposited onto lyophilized amniotic membranes by extrusion layer by layer. Although the shape of the in-vitro-cultured CECs was atypical, corneal transparency improved in the rabbit model at 2 weeks and approached complete transparency at 4 weeks postoperatively. In this study, additional biomaterial (the amniotic membrane) was used as a basis for printing. The goal of developing technology in the future should be to transplant scaffolds for bioink substrates only.

There have been studies that have reported inducing corneal limbal stem cells to differentiate into endothelial cells through specific bioinks using 7.5% GelMA and 2.5% hyaluronic acid methacryloyl (HAMA), as well as 7.5% acryloyl collagen and 25% poly (ethylene glycol) diacrylate (PEGDA) substrates as bioinks. This experiment induced the differentiation of primary human corneal stromal cells and corneal limbal stem cells into corneal epithelial cells and endothelial cells, respectively [88]. Some scholars have studied the technology system for efficiently inducing human embryonic stem cells (hESCs) to differentiate into corneal endothelial cells, and have achieved preliminary success in large animal models [89]. Therefore, it is presumed that CETE can be created via bioinks, where corneal endothelial cells can be obtained by promoting stem cell differentiation [90].

The technical challenges of preserving cell survival and construct building are all part of the intricate process of bioprinting, which also involves the choice of print kinds, materials, cell types, and other elements. In addition to conventional extrusion bioprinting, other methods like digital light processing printing (DLP), inkjet bioprinting, stereolithography apparatus (SLA), and laser-assisted bioprinting are being developed and updated. The use of DLP combined with extrusion printing has the potential to overcome the drawbacks of existing technologies, including slow speed and poor resolutions, while ensuring cell viability, high yield, and a superior optical performance [91,92].

### 7.3. Spin Coating Technology

Spin coating is a method used to deposit uniform film on a flat substrate. Usually, a small amount of coating material is applied to the center of the substrate while rotating at a low speed or without rotation. Subsequently, the substrate rotates at a speed of up to 10,000 rpm and diffuses the coating material through centrifugal force. Nanoscale-thick thin films can be prepared using spin coating technology.

Some scholars have suggested to take full advantage of spin coating technology to assist in the preparation of CETE. The scaffold can be prepared by dividing it into a two-layer structure. The structural layer can be prepared by spin coating, thereby achieving the preparation of the thinnest film. In contrast, the cell interaction layer can be fabricated by solvent casting, and topographical cues can be added using textured molds. The combination of several techniques can ensure the development of a thin, transparent, robust, and permeable scaffold that allows for the best possible cell response. Hoorick et al. reported a CETC scaffold using PDLLA and cross-linkable gelatins that was ultrathin (<1 μm), highly transparent (>90%), had a good mechanical strength, was semi-permeable, and had high biological potential [50].

### 7.4. Other Promising Technologies

With the advancement of tissue engineering and various bioprinting techniques, many interesting technological innovations have been spawned. Two-photon polymerization is a “nano-optical” 3D printing technology [93]. Two-photon polymerization has the benefit over traditional single-photon polymerization in that it can cure deep resin placement with sub-micron accuracy, enabling the printing of precise 3D objects in a variety of shapes. Nanoimprinting is a technology that transfers the microstructure on the template to the material to be processed with the aid of a photoresistor. Nanoimprinting lithography can not only create high-quality images with a resolution of 5 nm or even less, but also offers the benefit of a relatively simple procedure, high production capacity, low cost, and reusable imprinting templates [94].

Electrostatic direct writing technology extrudes fibers by melting the material and stretching them in an electrostatic field, allowing the originally thicker fibers to stretch and become finer. After dispersion and solidification, nanomaterials can be prepared. Compared to conventional cellular electrospinning, some studies have reported that electrostatic direct writing can be better deposited at the specified location to minimize loss. Moreover, the number of cells in electrostatic direct writing fibers is significantly higher than that in conventional cellular electrospinning due to dense fiber deposition. In terms of cell viability, electrostatic direct writing fibers are better than electrospinning, and their mechanical properties are closer to natural tissues [95].

These techniques can not only produce a specific bulk morphology but also help to achieve a complex, high-precision surface topography. It is possible to create uniform and optimal CETE scaffolds with the aid of these promising procedures.

## 8. Conclusions

In this review, we discuss innovative biomaterials and technologies for developing CETE scaffolds. The scaffold-based CETE is an ideal substitute for donor-derived grafts, and great progress has been made in this field. Transparency, permeability, mechanical properties, biocompatibility, and the ability to maintain the differentiated state of CECs are the key characteristics required for corneal endothelium substrates. The substrate materials that have been studied consist of natural biomaterials, synthetic polymers, and semi-synthetic polymers. Natural biomaterials have good biocompatibility and cell adhesion, while synthetic polymers can be artificially controlled to produce scaffolds with predictable structures and physicochemical properties. Semi-synthetic polymers seem to meet all needs simultaneously, enhancing both the physical and biological properties of the scaffold. Cutting-edge materials such as polypeptide hydrogel, injectable hydrogel, and functional nanomaterials have their own unique advantages and are worth trying in the future.

The scaffold’s surface topography, simulating the ECM microenvironment, provides physical signals for CECs that are crucial for their growth. Additionally, scaffold should be supplemented with bioactive molecules via drug delivery systems to promote the morphology and function of CECs. Advanced technologies including electrospinning, bioprinting, and spin coating have shown their superior potential in the process of scaffold preparation. Future studies will focus on combining multiple technologies to achieve a high-precision, uniform, and mass production of CETE scaffolds.

On a final note, despite the fact that many designs have been proven to promote CECs growth, the mechanism of this effect is still unclear. The underlying molecular mechanisms and signaling pathways require further study. At present, there are still technical difficulties in standardizing the parameters of the scaffold. It is necessary to evaluate the scaffold–cells interaction through physiological mechanics and biological platforms, and then to establish criteria for evaluating biomechanical properties. Despite both biodegradable and non-biodegradable materials being able to be used as scaffolds, the degradation rate of biodegradable scaffolds needs to be matched with the regeneration rate of DM to support CECs. However, there are currently few investigations between the two. These questions should be answered in the future, and clinical trials and industrialization should be carried out with caution.

## Figures and Tables

**Table 1:** Summary of the advantages and limitations of each material for scaffold-based corneal endothelium tissue engineering.

Category	Type	Advantages	Limitation	In Vivo Study	Use with hCECs	Reference
Natural tissue	Decellularized corneal stroma	Appropriate mechanical properties, natural recognition signals	Shortage of donors, xenoantigen causes immune rejection, the risk of infection transmission	Rat	Yes	[23]
	Descemet’s membrane	Mimics the natural ECM environment	Too thin to operate, the risk of infection transmission	Cat	Yes	[24]
	Amniotic membrane	Low immunogenicity, biocompatibility, widely used in ophthalmology	Semi-transparency, shortage of donors, heterogeneous, insufficient mechanical strength, unpredictable biodegradation rates, potential for granulomatous reactions, the risk of contamination and infection transmission	Rabbit, cat, monkey	Yes	[25,26,27]
	Decellularized lens capsule membrane	Transparency, similar to Descemet’s membrane	Shortage of donors, small diameter	Pig	Yes	[28]
	Decellularized fish scales	Transparency, good mechanical strength, availability, biocompatibility	Poor cell proliferation and adhesion	Rabbit	Yes	[29,30]
Natural polymers	Collagen	Transparency, desirable biodegradability and biocompatibility	Insufficient mechanical strength	Rabbit	Yes	[31]
	Gelatin	Transparency, flexible, cost-effective, availability, desirable biodegradability and biocompatibility	Insufficient mechanical strength	Monkey, rabbit	Yes	[10,32]
	Hyaluronic acid	Biocompatibility	Rapid dissolution in a liquid environment, insufficient mechanical strength	Rabbit	Yes	[32]
	Silk proteins	Low immunogenicity, good transparency, non-cytotoxic	Insufficient mechanical strength, fragile	Rabbit	Yes	[33,34,35]
	Chitosan	Good biodegradability and biocompatibility	No in vivo studies, insufficient mechanical strength, inflammation	No	No	[36]
Synthetic polymers	PLGA	Biocompatible, good mechanical strength	No in vivo studies, faster degradation rate resulting in a more acidic pH in the culture media	No	No	[37]
	PEG	Transparency, good mechanical strength, biocompatibility	No reports on biodegradation	Sheep	No	[9]
	PVDF	Biocompatibility, good mechanical strength, chemically inert	No in vivo studies, no reports on biodegradation	No	No	[38]
Semi-synthetic polymers	GelMA+	Increased mechanical strength, good temperature-sensitive properties, biocompatibility, printability	Expensive, long production process	Rabbit	Yes	[39]
	Chitosan and PEG	Biodegradable, increased mechanical strength, transparency	No in vivo studies	No	No	[40]
	Chitosan and PCL	Biodegradable, increased mechanical strength	No in vivo studies	No	No	[41]

hCECs, human corneal endothelial cells; ECM, extracellular matrix; PLGA, poly (lactic-co-glutamic acid); PCL, polycaprolactone; PEG, polyethylene glycol; PVDF, polyvinylidene fluoride; GelMA, methacryloyl gelatin.

**Table 2:** Summary of the recent published literature on emerging technologies for CETE scaffold preparation.

Technology Type	Year	Substrate	Advantage	Limitation	In Vivo Study	Use with hCECs
Electrospinning	2020	Silk fibroin nanofibers [80]	Bead-free and continuous nanofibers, homogeneity and high growth of cells, greater Young’s modulus compared to natural cornea	No result of transparency, no in vivo study	No	No
	2021	PCL, PCL/collagen, PCL/gelatin, PCL/chitosan [81]	Enhanced the properties of electrospun nanofibrous scaffolds, increased cell viability, no cytotoxic threat	No result of transparency, no in vivo study, fiber diameters (174 ± 119 nm) larger than collagen (25–35 nm)	No	Yes
	2021	PCL [82]	Sufficiently high transmission values were only obtained below 5 μm, whereby scaffolds with thinner fiber diameters (35nm) showed a higher light transmission	No results of properties except light transmission, no in vivo study	No	No
Bioprinting	2021	Poly-ε-lysine and gellan gum [83]	Three-dimensional structures with a high resolution by reactive inkjet printing (RIJ), unique pattern surface, good cyto-compatibility	Transparency of 80%, no in vivo study	No	Yes
Spin coating	2020	Poly (D, L-lactic acid) and cross-linkable gelatins [50]	Ultrathin (<1 μm), highly transparent (>90%), good mechanical strength, semi-permeable, high biological potential	No in vivo study	No	Yes
